# Germline encoded residues dominate the interaction of a human monoclonal antibody with decorin binding protein A of *Borrelia burgdorferi*

**DOI:** 10.3389/fimmu.2025.1611828

**Published:** 2025-07-07

**Authors:** Michael J. Rudolph, Beatrice M. Muriuki, Yang Chen, David J. Vance, Clint Vorauer, Carol Lyn Piazza, Grace Freeman-Gallant, Rachel M. Golonka, Gianna Mirabile, Miklos Guttman, Lisa A. Cavacini, Nicholas J. Mantis

**Affiliations:** ^1^ New York Structural Biology Center, New York, NY, United States; ^2^ Department of Medicine, University of Massachusetts Chan Medical School, Worcester, MA, United States; ^3^ Division of Infectious Diseases, Wadsworth Center, NY State Department of Health, Albany, NY, United States; ^4^ Department of Biomedical Sciences, University at Albany, Albany, NY, United States; ^5^ Department of Medicinal Chemistry, University of Washington, Seattle, WA, United States

**Keywords:** human, mouse, lyme antibodies, vaccines, *Borrelia (Borreliella) burgdorferi*

## Abstract

During the course of Lyme disease, humans mount a robust and sustained antibody response against dozens of *Borrelia burgdorferi* outer surface lipoproteins. Identifying which antibodies are associated with spirochete clearance and disease resolution is of paramount importance in therapeutic development. In this study, we describe the isolation and structural characterization of a human monoclonal antibody (MAb) against decorin binding protein A (DbpA), one of the most immunogenic of *B. burgdorferi*’s outer surface proteins. High-resolution epitope mapping by HDX-MS and X-ray crystallography revealed that F945 associates with a lateral face of DbpA in a side-on orientation without obstructing resides associated with DbpA’s ability to bind components of the extracellular matrix. The structure of the DbpA-F945 Fab complex revealed an outsized role for variable light chain (V_L_) germline encoded residues in mediating DbpA interactions. In fact, the majority of the critical contacts between F945 and DbpA involved V_k_1–33 germline encoded residues, suggesting that certain human B cell receptors (BCR) may be preconfigured to recognize DbpA and therefore have a lower threshold for B cell activation and clonal development. Passive administration of F945 IgG was not sufficient to protect against *B. burgdorferi* in a mouse model of needle infection, although these experiments do not rule out a role for F945 in influencing *B. burgdorferi* tissue tropism and/or retention within specific niches. Nonetheless, it is tempting to speculate that F945 represents a class of DbpA antibodies with value in Lyme disease diagnostics, but that may not contribute to *B. burgdorferi* clearance or disease resolution in humans.

## 1 Introduction

The spirochete, *Borrelia burgdorferi* sensu latu, is the primary etiological agent of Lyme disease (LD), an increasingly common tick-borne illness in the United States and Europe that afflicts individuals of all ages. In the absence of antibiotic intervention, *B. burgdorferi* can disseminate over the course of days and weeks from the skin to distal tissues with clinical manifestations spanning neuroborreliosis, carditis, and Lyme arthritis (1). *B. burgdorferi* infection is accompanied by a robust antibody response directed against the dozens of outer surface proteins that are expressed by the bacterium upon entry and dissemination in mammalian hosts, including humans (2, 3). The rapid resolution of Lyme disease is associated with a robust B cell response and concomitant appearance of antibodies against a range of targets (4). Sorting out which antibody populations drive spirochete clearance from distal tissues and disease resolution is of paramount importance when considering the development of Lyme disease therapeutics (5).

One of the most immunogenic of *B. burgdorferi*’s outer surface proteins is decorin binding protein A (DbpA; BBA24; UniProt O50917). DbpA is a helical lipoprotein of ~19 kDa that promotes *B. burgdorferi* attachment to connective tissues and components of the extracellular matrix (ECM), including glycosaminoglycans (GAGs) such as decorin, dermatan sulfate, and heparin (6–15). By virtue of its ability to adhere to GAGs, DbpA influences *B. burgdorferi* tropism for specific tissues and cell types (13, 16). DbpA is expressed early during infection and stimulates the onset of antibodies, even in the absence of CD4 T cell help (17, 18). The humoral response to DbpA arises rapidly following infection and can persist for months, even following antibiotic treatment (19, 20). In fact, the B cell response is so robust in humans that anti-DbpA antibodies are considered diagnostic of Lyme disease (21–27).

Although DbpA is one of the most antigenic of *B. burgdorferi*’s lipoproteins and proposed to play an important role in spirochete tissue tropism, the impact of DbpA antibodies on infectivity and disease course remains enigmatic. On the one hand, preexisting antibodies to DbpA as a result of passive or active immunization have been shown to protect mice against *B. burgdorferi* needle infection (9, 17, 28–31). DbpA antibodies have also been implicated in disease remission, as administration of hyperimmune DbpA antisera to C3H-scid mice within days after spirochete challenge reduced the prevalence and severity of *B. burgdorferi*-induced arthritis and carditis (17). On the other hand, Hagman and colleagues reported that DbpA vaccination in mice does not protect mice from tick-mediated *B. burgdorferi* infection, nor does it appear to impact spirochete clearance (20). In fact, evidence suggests that DbpA expression continues unabated in the face of a robust antibody response (20, 32, 33).

Despite being a primary target of the humoral immune response in LD, virtually nothing is known about the specific epitopes on DbpA recognized by human LD patients. Moreover, considering the conflicting reports from mouse studies, it is difficult to know how DbpA antibodies modulate the course and severity of Lyme disease in humans, where the detailed interactions between DbpA and components of the adaptive immune response remains largely unexplored beyond surveys of linear B cell epitopes for the sake of diagnostic assays (21–27). We recently analyzed ~270 human serum samples and identified several linear epitopes that may be associated with functional activities, including a flexible linker between α-helix 1 and 2, and the C-terminus (34). However, those were strictly linear epitopes and no investigation into conformational epitopes has been reported. In this study, we now describe the isolation and structural characterization of a DbpA-specific human monoclonal antibody (MAb) and uncover novel insights into the nature of the humoral response to this highly immunoreactive protein in Lyme disease.

## 2 Materials and methods

### 2.1 Cloning, expression, and purification of DbpA

The PCR amplicon encoding *B. burgdorferi* B31 DbpA (residues 26 to 188; UniProt O50917) was subcloned into the pNYCOMPS expression vector that contained a C-terminal deca-histidine tag. Cloning was performed using a standard ligase independent cloning. Recombinant DbpA (rDbpA) was expressed in Shuffle T7 *E. coli* cells. The transformed bacteria were grown at 37°C in TB medium and induced at 20°C at an OD_600_ of 0.6 with 0.1 mM IPTG for ~16 h. After induction, cells were harvested and resuspended in 20 mM HEPES [pH 7.5] and 150 mM NaCl. The cell suspension was sonicated and centrifuged at 30,000 x *g* for 30 min. After centrifugation, the protein-containing supernatant was purified by nickel-affinity and size-exclusion chromatography on an AKTAxpress system (GE Healthcare), which consisted of a 1 mL nickel affinity column followed by a Superdex 200 16/60 gel filtration column. The elution buffer consisted of 0.5 M imidazole in binding buffer, and the gel filtration buffer consisted of 20 mM HEPES [pH 7.5], 150 mM NaCl, and 20 mM imidazole. Fractions containing DbpA were pooled and subject to TEV protease cleavage (1:10 weight ratio) for 3 h at room temperature to remove respective fusion protein tags. The cleaved protein was passed over a 1mL Ni-NTA agarose (Qiagen) gravity column to remove TEV protease, cleaved residues, and uncleaved fusion protein.

### 2.2 Generation of DbpA-specific human monoclonal antibody F945

Peripheral blood B cells from a clinically confirmed patient recovering from Lyme disease were isolated using RosetteSep (Stem Cell Technology, Vancouver, Canada) following the manufacturer’s instructions. All studies were approved by the UMass Chan Institutional Review Board (IRB). Enriched B cells were stimulated with a mitogen cocktail for 48 h before being fused with a human-mouse myeloma cell line (HMMA2.5) (35, 36). Hybridoma supernatants were screened for reactivity with Lyme antigens by ELISA and those producing DbpA-specific antibodies were selected for sequencing. The F945 human MAb was characterized for additional analysis as stated below. The F945 V_H_ and V_L_ sequences are provided in **Supplementary Table S1** and submitted to GenBank accession numbers (VH) PV083662 and (VL) PV083663.

### 2.3 Dot blot analysis

*B. burgdorferi* strain GGW941, a derivative of strain B31 that carries a plasmid encoding *rpoS* under control of the IPTG inducible promoter (37), was cultured in BSK-II medium (minus gelatin) plus gentamycin (50 µg/ml) at 37 °C with 5% CO_2_ to mid-log phase, then treated with 0.05 or 0.50 mM IPTG for an additional ~48 h. Cells were then collected by centrifugation (3,300 x *g* for 10 min) and resulting pellets were stored at -20 °C. When needed, bacterial cells (1 x 10^7^ equivalent) were thawed, resuspended in PBS and then spotted as 10-fold serial dilutions on 0.2 µm nitrocellulose membrane. As a control, serial dilutions of recombinant DbpA (1 µg) were spotted alongside. Membrane were probed with F945 (0.1 μg/mL) and developed using ECL as described (38).

### 2.4 Flow cytometry

*B. burgdorferi* strain GGW941 was cultured in BSK II, as described above, and stored at -80 °C in fresh medium containing 20% glycerol at a cell density of 1 x 10^8^ cells/ml. When needed, the frozen glycerol stocks were thawed, washed with PBS, resuspended in gelatin- and phenol-free BSK-II and allowed to recover for 2 h at room temperature. Cells were then incubated with F945 (10 µg/ml) or Sal4 IgG as isotype control (provided by ZabBio, San Diego, CA) (39). Flow cytometry was performed using a FACS Calibur, as described (38). Histogram analysis was done using FlowJo software (Ashland, OR)

### 2.5 ELISA

High-binding 96 well ELISA plates (Costar) were coated overnight at 4°C with rDbpA. After blocking the plates (Blocker BSA, Thermo Fisher Scientific), serially diluted antibodies were allowed to bind to the plates for 30 min. Antibody was detected using horseradish peroxidase (HRP)-conjugated goat anti-human IgG antibodies (Southern Biotech, Birmingham, AL) after incubation for 30 min. The assay was developed using 3,3’,5,5’-tetramethylbenzidine reagents (TMB) solution (SeraCare, Milford, MA). The reaction was stopped after 5 min using 1 M phosphoric acid and the absorbance of each well measured at 405nm or 450nm on a spectrophotometer using BioTek Gen5. EC_50_ values were calculated using GraphPad Prism 8.0 software. All experiments were performed in triplicate.

### 2.6 Antibody sequencing, cloning, and expression

Total RNA was extracted from positive hybridoma cell clones using RNeasy kit (Qiagen, Germany) following manufacturer’s instructions. RNA was converted to cDNA using superscript III first-strand synthesis system (Invitrogen, USA). Amplification of the heavy- or light- chain sequences were performed using a superscript III one-step RT-PCR kit (Invitrogen, USA) and human V_H_ or V_L_ genes (forward primers) and constant region (reverse primers) (40). The PCR products were gel purified using QIAquick^®^ gel extraction kit (Qiagen, Germany), and the antibody variable regions Sanger-sequenced (Azenta). The raw FASTA format data were annotated for V(D)J germline genes using reference V(D)J sequences from the IMGT database (41).

F945 IgG was generated by cloning the immunoglobulin variable heavy- and variable light-chain genes (VH/VL) into pcDNA3.1 in-frame with human IgG1 and human kappa chain backbones, respectively (Genscript, New Jersey). Expi293 cells were transiently transfected according to the instructions (ThermoFisher Scientific, USA). After five days of culture, the supernatants containing the secreted antibodies were harvested, clarified and purified using protein A chromatography. The purified antibodies were buffer exchanged into PBS and stored at 4°C.

### 2.7 Hydrogen-deuterium exchange

Ten microliters of rDbpA_B31_ (8 µM) in PBS or with 1.5-fold excess of F945 IgG were diluted into 90 µL of deuterated PBS buffer (20 mM phosphate, 150 mM NaCl, 0.02% sodium azide, 1 mM EDTA pH* 7.54, 85%D final) containing 0.2 nM bradykinin and incubated for 3 sec, 1 min, 30 min, or 20 h at 21°C. Each starting stock also included a mixture of imidazolium compounds to serve as exchange reference standards (42). At the desired time point the sample was rapidly mixed with an equal volume of ice cold 8 M urea, 0.2% formic acid and 0.1% trifluoroacetic acid (TFA) for a final pH of 2.5. Samples were then frozen on ethanol/dry ice and stored at -80°C until LC-MS analysis. Undeuterated samples were prepared the same way but with undeuterated buffer for each step.

Samples were thawed at 5°C for 4 minutes and injected using a custom LEAP robot integrated with an LC-MS system (43). The protein was first passed over a Nepenthesin II column (2.1 x 30 mm; AffiPro, Vestec, Czech Republic) at 400 µL/min for inline digestion with the protease column held at 20°C. Peptides were then trapped on a Waters XSelect CSH C18 trap cartridge column (2.1 x 5 mm 2.5 µm) and resolved over a CSH C18 column (1 x 50 mm 1.7 µm 130Å) using linear gradient of 5 to 35% B (A: 0.1% FA, 0.025% TFA, 5% ACN; B: ACN with 0.1% FA) over 10 min and analyzed on a Waters Synapt G2-Si with ion mobility enabled. A series of washes over the trap and pepsin columns was used between injections to minimize carry-over as described previously (43). Data dependent MS/MS acquisition was performed on an undeuterated sample with the same LC conditions using rapid CID and HCD scans on a Thermo Orbitrap Ascend and processed in Byonic (Protein Metrics, Cupertino, CA) with a score cutoff of 150 to identify peptides. Deuterium incorporation was analyzed using HDExaminer v3 (Trajan Scientific and Medical, Ringwood, Australia). Raw data is available through the PRIDE ProteomeXchange repository accession PXD062826 (44).

### 2.8 Crystallization and data collection

Crystals were grown by sitting drop vapor diffusion using a protein to reservoir volume ratio of 1:1 with total drop volumes of 0.2 μl. The F945 Fab was generated by papain digestion followed by affinity depletion of the Fc fragment by Protein A FPLC chromatography. After purification, Fab F945 was complexed with DbpA in a 1:1 stoichiometry, then concentrated to 10 mg/ml for crystallization trials. Crystals of the F945 Fab-DbpA complex were produced at 22°C using a crystallization solution containing 200 mM ammonium sulfate and 20% PEG 6K. Crystals were flash frozen in liquid nitrogen after a short soak in the appropriate crystallization buffers supplemented with 25% ethylene glycol. Data were collected at the 19-ID beamline at the National Synchrotron Light Source II, Brookhaven National Labs. All data was indexed, merged, and scaled using HKL2000 (45), then converted to structure factor amplitudes using CCP4 (46).

### 2.9 Structure determination and refinement

The F945 Fab-DbpA complex structure was solved by molecular replacement using Phaser (45). Molecular replacement calculations were performed independently using the V_L_ and C_L_ domain and with the V_H_ and C_H_1 domain coordinates of the EY6A Fab (PDB ID: 6ZCZ) as the search model for the F945 Fab in F945-DbpA complex within the asymmetric unit. The DbpA coordinates (PDB ID:4ONR) were then used as the search model for the DbpA monomer in the F945-DbpA complex. The resulting phase information from molecular replacement was used for some manual model building of the F945-DbpA structure using the graphics program COOT (47) and structural refinement employing the PHENIX package (48). Data collection and refinement statistics are listed in **Supplementary Table S1**. The Protein Data Bank code (PDB ID) for the F945-DbpA structure is 9BQW (http://www.rcsb.org/pdb/). Molecular graphics were prepared using PyMOL (Schrodinger, DeLano Scientific LLC, Palo Alto, CA).

### 2.10 Binding affinity determination by BLI

Biotinylated N-terminal avi-tagged rDbpA (2 µg/mL) diluted into PBS containing 2% w/v BSA was captured onto streptavidin biosensors (#18-5019, Sartorius, Goettingen, Germany) then exposed to a 2-fold dilution series of F945 (1.56 to 100 nM) for 10 min to allow association. The sensors were then dipped into buffer for 30 min to allow dissociation. As a background drift control (i.e. dissociation of ligand from sensor), we included an eighth sensor loaded with biotinylated DbpA that was not exposed to MAb. The values associated with the drift control where then subtracted from other sensor data to correct for loss of DbpA-streptavidin sensor. Data was captured on an Octet RED96e Biolayer Interferometer (Sartorius) using the Data Acquisition 12.0 software. Binding kinetics were determined using Data Analysis HT 12.0 software and fit to a 1:2 bivalent analyte model.

### 2.11 Passive immunization studies in a mouse model of *B. burgdorferi* infection

Mouse experiments were conducted with prior approval from the Wadsworth Center’s Institutional Animal Care and Use Committee (IACUC). The kinetics of *B. burgdorferi* infection following needle injection are described (49). Female BALB/c mice aged ~8 weeks (Taconic Biosciences, Germantown, NY) were allowed to acclimate in the vivarium for one week before the start of the experiment. On experiment day -1, mice were injected intraperitoneally (IP) with 120 µg of mAb in 200 µL PBS. The following day (study day 0), mice were challenged with 10^5^ *B. burgdorferi* strain B31 A4 by intradermal injection. On day 21, the mice were euthanized, and blood was collected via cardiac puncture. A newly described human anti-OspC mAb called F946 was used as a positive control for these studies (M. Rudolph, D. Vance, L. Cavacini, N. Mantis, *manuscript in preparation*).

### 2.12 *B. burgdorferi* antigen multiplex microsphere immunoassay

Recombinant *B. burgdorferi* antigens or peptides were coupled to MagPlex-C microspheres, as described (50). Diluted microspheres (50 μL) and diluted sera or MAb (50 μL) were combined in black, clear-bottomed, non-binding, chimney 96-well plates (Greiner Bio-One, Monroe, NC) and incubated at RT for 1 h in a tabletop shaker. Plates were washed three times using a magnetic separator and wash buffer (1 x PBS, 2% BSA, 0.02% Tween-20, 0.05% sodium azide, pH 7.4) then probed with goat anti-mouse IgG, human-ads-PE (Southern Biotech) diluted 1:500. Plates were analyzed using a FlexMap 3D (Luminex Corporation). To establish reactivity cutoffs for seroconversion in passive immunization studies, the average median florescent intensity (MFI) of wells containing naïve mouse sera was multiplied by six. MFIs for each mouse serum sample were divided by the antigen-specific reactivity cutoffs yielding an index value. An index value greater than 1 is indicative of reactivity above background for the given antigen.

### 2.13 Statistics

Statistical analysis was carried out using GraphPad Prism 8.0 software. The specific tests, sample sizes, and number of biological and technical replicates are indicated in the Results, tables and/or figure legends.

## 3 Results

### 3.1 Isolation of a human monoclonal antibody specific for DbpA

**Figure 1:**
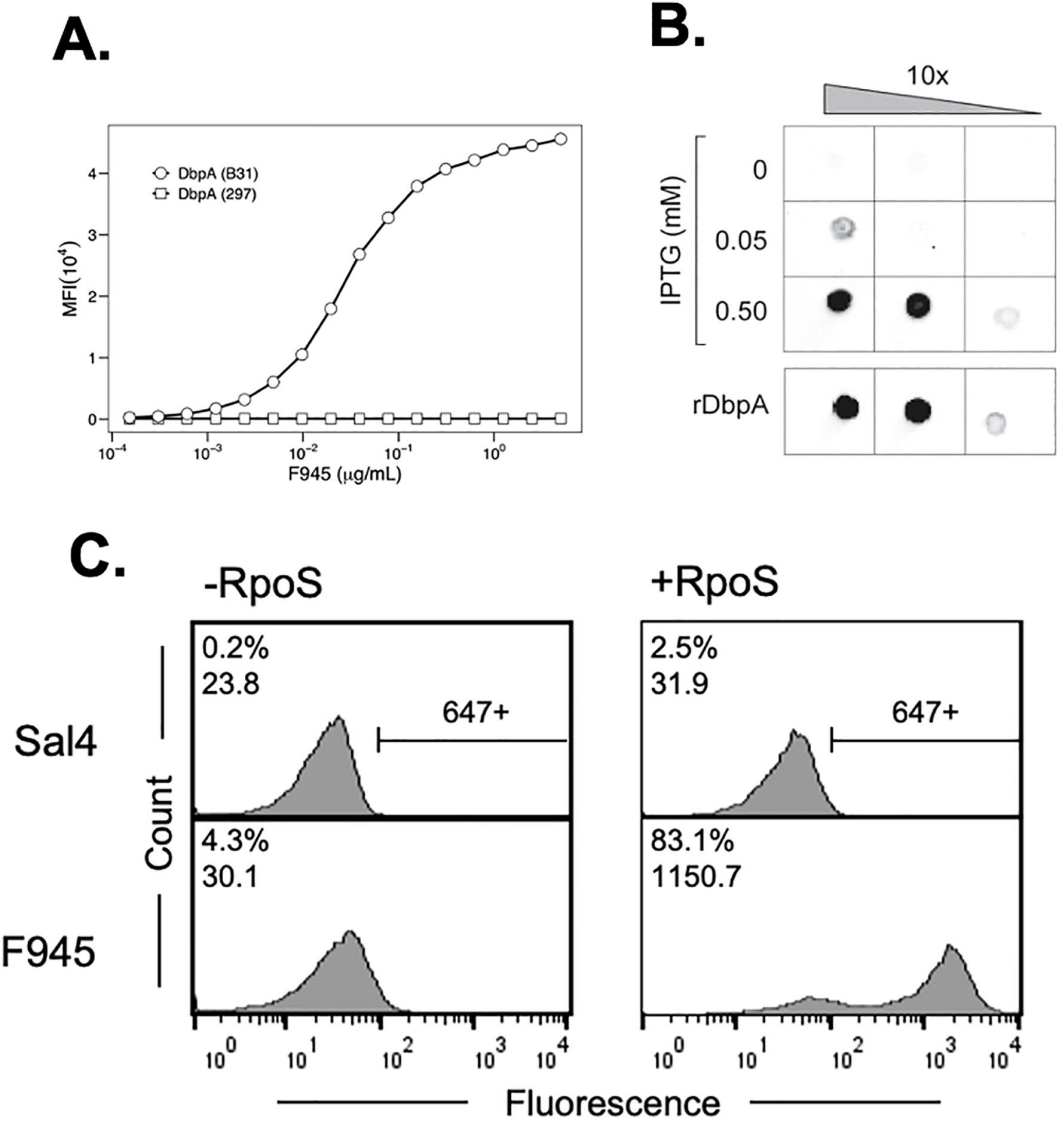
Reactivity of F945 IgG with recombinant and native DbpA_B31_. **(A)** Dose-dependent reactivity of F945 IgG with recombinant DbpA_B31_ (open circles) but not recombinant DbpA_297_ (open squares), as determined by multiplex immunoassay. MFI, median fluorescence intensity. **(B)** F945 IgG reactivity to native DbpA_B31_ by dot blot. Ten-fold serial dilutions (left to right) of *B*. *burgdorferi* strain GGW941 treated with indicated amounts of IPTG to stimulate the production of RpoS and DbpA was spotted onto nitrocellulose membrane, then probed with F945 IgG, as described in the Materials and Methods. Recombinant DbpA_B31_ was spotted separately as a control. **(C)** Flow cytometric analysis of F945 IgG reactivity with the surface of *B*. *burgdorferi* strain GGW941 not treated (-RpoS; left panels) or treated (+RpoS; right panels) with IPTG to induce RpoS and DbpA expression. The *Salmonella*-specific mAb, Sal4 IgG, was used as an isotype control (top panels). The histograms plot number of cells (“count”) versus fluorescence intensity (“fluorescence”). The numbers in the top left corner of each box represent positive events (% of total) and MFI. The horizontal bar to the right of the histograms in the top panels indicate threshold for positivity. Each panel depicts a single experiment representative of at least two biological replicates.

B cells isolated from a convalescent Lyme disease patient (“F945”) were fused with a HeteroMyeloma human-mouse cell line (HMMA 2.5) and the resulting hybridomas were screened for reactivity with rDbpA_B31_. Of the 576 hybridoma screened, we identified one that continuously secreted IgG reactive with rDbpA_B31_. The V_H_ and V_L_ variable regions from the single-cell cloned hybridoma were PCR amplified and ligated in-frame into human IgG1 and kappa light chain expression vectors, respectively. The predicted V gene usages were IGHV3–30 and IGKV1-33, based on IMGT alignments (51). The resulting human recombinant Mab, simply referred to as F945 from this point forward, reacted with recombinant DbpA_B31_ by a microsphere immunoassay (MIA) (**Figure 1A**). Interestingly, F945 did not react with recombinant DbpA from *B. burgdorferi* strain 297, even though DbpA_297_ shares ~90% amino acid identity with DbpA_B31_ (**Figure 1A**; **Supplementary Figure S1**) (31). Binding kinetic determinations by BLI indicated that F945’s apparent affinity (K_D_) for rDbpA_B31_ is ~ 4 nM (**Supplementary Figure S2**).

We next examined the ability of F945 to react with native DbpA_B31_. As DbpA is normally expressed at very low levels by *B. burgdorferi* grown in culture, we employed a derivative of strain B31 called GGW941 that carries a plasmid encoding *rpoS* under control of the IPTG inducible promoter as a means to stimulate DbpA expression in trans (37, 52). By dot blot analysis, F945 reacted with GGW941 treated with 0.05 or 0.5 mM IPTG (resulting in RpoS and DbpA expression) but not with untreated cells (**Figure 1B**). To quantitate F945 binding on the spirochete surface, live cultures of GGW941 treated or not with IPTG were thawed from glycerol working stocks and probed with F945 followed by an Alexa 647-labeled secondary antibody. By flow cytometry, F945 labeling was markedly higher with GGW941 cells that had been treated with IPTG than the untreated cells, as evidenced by both the number of cells labeled (~80% versus ~4.0% labeled) and the magnitude of staining (1150 versus 30 MFI) (**Figure 1C**). The observed reactivity was specific to F945, as an isotype control antibody against *Salmonella* LPS displayed only marginal cell staining (0.2-2.5%). Thus, F945 recognizes native DbpA.

As an aside, optimal surface reactivity of live *B. burgdorferi* with F945 was observed under conditions where the bacterial outer membrane was lightly permeabilized, by either being freshly thawed from glycerol working stocks, or when actively growing cells were treated with a low percentage (0.05-0.5%) of Tween-20 (**Supplementary Figure S2B**). One interpretation of these observations is that F945’s epitope is partially occluded by or buried within the bacterial outer surface and that mild perturbation of the membrane enables antibody accessibility. Indeed, the idea that antibodies may have limited accessibility to DpbA has been suggested by others (20).

### 3.2 Localization of F945’s epitope by HDX-MS

**Figure 2:**
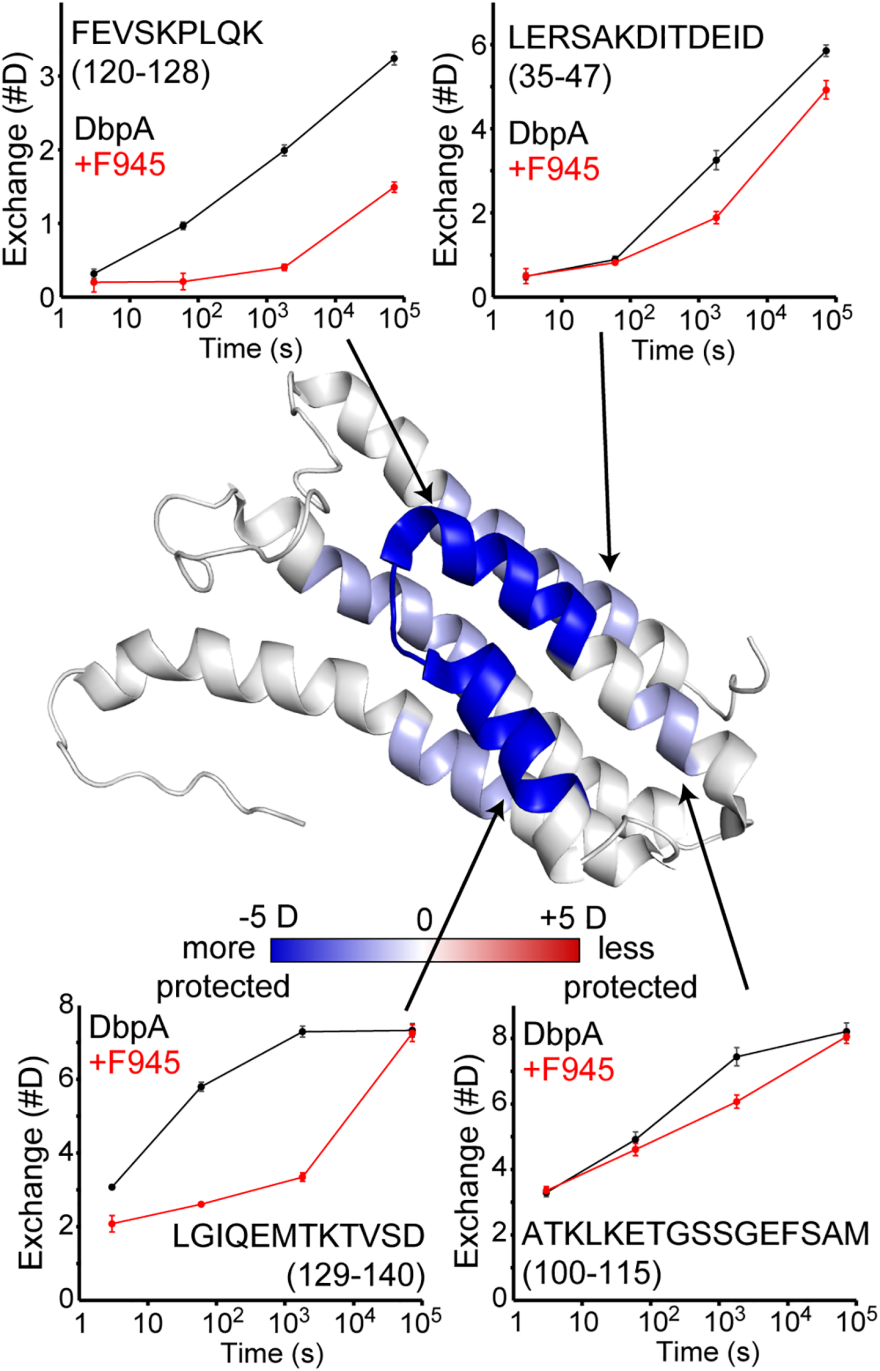
Localization of F945’s epitope on DbpA by HDX-MS. HDX changes upon binding mAb F945 are plotted on the structure of DbpA (PDB: 2LQU) with regions becoming more protected (blue) or more exposed (red) based on the sum difference across all time points. Deuterium uptake plots for unbound DbpA (black) and the DbpA-F945 complex (red) are shown for selected regions as indicated by the arrows. Error bars represent standard deviations from quadruplicate measurements. The full HDX-MS profile and peptide coverage is shown in **Supplementary Figure S3**.

The fact that F945 recognizes DbpA_B31_ but not the closely related DbpA_297_ indicates that F945’s epitope likely corresponds to a region of non-identity. To localize F945’s epitope, we employed hydrogen/deuterium exchange mass spectrometry (HDX-MS) following protocols employed with other *B. burgdorferi* lipoproteins (37, 53–56). Digestion of rDbpA_B31_ with the aspartic protease, nepenthesin II, yielded 47 observable peptides covering rDbpA_B31_ with an average redundancy of 3.3 (**Supplementary Figure S3**). Using this enzyme, we then compared the HDX-MS profile of rDbpA in the absence and presence of 1.5-fold molar excess of F945 (**Figure 2**). In the presence of F945, there was strong protection of two peptides corresponding to residues 120–128 and 129–140 and minor to moderate protection of two additional peptides corresponding to residues 35–47 and 100-115. When mapped onto the tertiary structure of DbpA_B31_ [PDB ID 2LQU], the two strongest protected peptides aligned with α-helices 3 and 4 and the intervening loop (loop 3-4) (**Figure 2**). The two moderately protective peptides corresponded to the core of α-helix 1 (residues 35-47) and portions of α-helix 2, loop 2–3 and the proximal region of α-helix 3 (residues 100-115). These results suggest that the F945’s epitope is focused along a lateral face of DbpA, principally involving α-helices 3 and 4.

### 3.3 X-ray crystal structure of DbpA-F945 Fab complex

**Figure 3:**
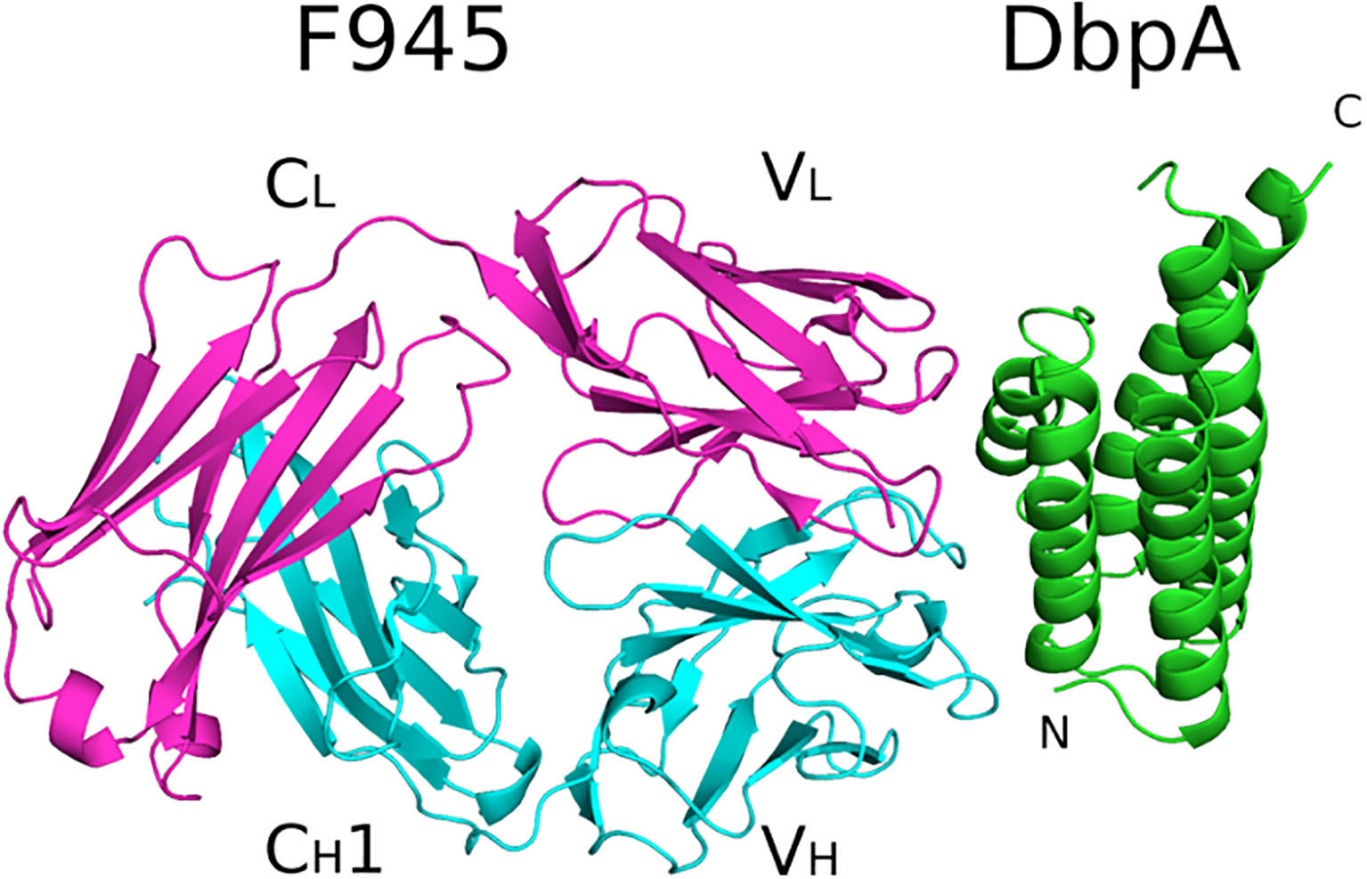
Structure of F945 Fab-DbpA complex. Structure of the Fab F945-DbpA complex with the Fab heavy chain (V_H_ and C_H_1) colored cyan, the light chain (V_L_ and C_L_) colored magenta, and both depicted as ribbon diagrams. DbpA is drawn as green ribbons. The V_H_, C_H_1, V_L_ and C_L_ domains (V_H_,V_L_) along with the N- and C-termini of DbpA (N,C) are labeled accordingly.

To resolve F945’s epitope in greater detail, we determined the X-ray crystal structure of F945 Fab bound to recombinant DbpA_B31_ at 2.5 Å resolution in the P2_1_2_1_2 space group (**Table 1**). Within the complex, the F945 Fab adopted the conventional antibody configuration with two heavy chain immunoglobulin domains (V_H_, C_H1_) and two light immunoglobulin domains (V_L_, C_L_). Each domain consisted of 7-10 β-strands organized into two β-sheets, forming a two-layered β-sandwich structure. All six CDRs (L1-L3, H1-H3) were situated on the same side of the molecule (**Figure 3**). DbpA_B31_ also assumed its expected tertiary conformation as a 5 α-helical bundle. Superimposition of unbound DbpA_297_ [PDB ID 4ONR] with DbpA_B31_ in complex with F945 Fab showed an RMSD of 0.6 Å, confirming the structural similarity between the two DbpA molecules (**Supplementary Figure S4**). The structure of DbpA_B31_ also revealed a sulfate ion bound within DbpA’s Lys pocket (Lys-82, Lys-163, Lys-170), the significance of which will be discussed below.

**Table 1:** Data associated with F945-DbpA complex.

Data Collection	
Complex	F945 FAb-DbpA_B31_
Space group	P2_1_2_1_2
Cell parameters: *a,b,c* (Å)	51.1, 86.5, 151.5
BNL Beamline	19-ID-E
Resolution range *^a,^* (Å)	50-2.55 (2.59-2.55)
wavelength (Å)	0.979
No. of reflections	41669
Average redundancy*^a^*	11.1 (5.9)
(*I*)/(δ)*^a^*	12.7 (0.9)
Completeness*^a^* (%)	99.3 (90.7)
*R*_merge_ *^a,b^* (%)	12.4 (79.6)
CC^1/2 a^ *^,c^*	(0.80)
Refinement	
Bragg spacings *^d^* (Å)	48.4-2.55 (2.66-2.55)
*R^d/^R*_free_ *^e^* (%)	20.2/24.6
No. of Protein atoms	4,305
No. of Waters	58
RMSD bond length (Å)	0.003
RMSD bond angle (°)	0.604
Ramachandran favored/allowed*^f^* (%)	97.5/100
**PDB ID**	**9BQW**

*^a^* Values in outermost shell are given in parentheses.

*^b^ R*_merge_ = (∑│I_i_ - <I_i_>│)/∑│I_i_ │, where I_i_ is the integrated intensity of a given reflection.

*^c^* CC_1/2_= (1+q^2^s_e_^2^/<I>2^-1^, where s_e_ denotes the mean error within a half-datase, CC_1/2_ is the correlation coefficient of two split data sets each derived by averaging half of the observations for a given reflection.

*^d^R* = ∑│|F*_o_* | - |F*_c_* |│/∑│|F*_o_* |│, where F*_o_* and F*_c_* denote observe and calculated structure factors, respectively.

*^e^R*_free_was calculated using 5% of data excluded from refinement.

^f^Calculated using Molprobity.

**Figure 4:**
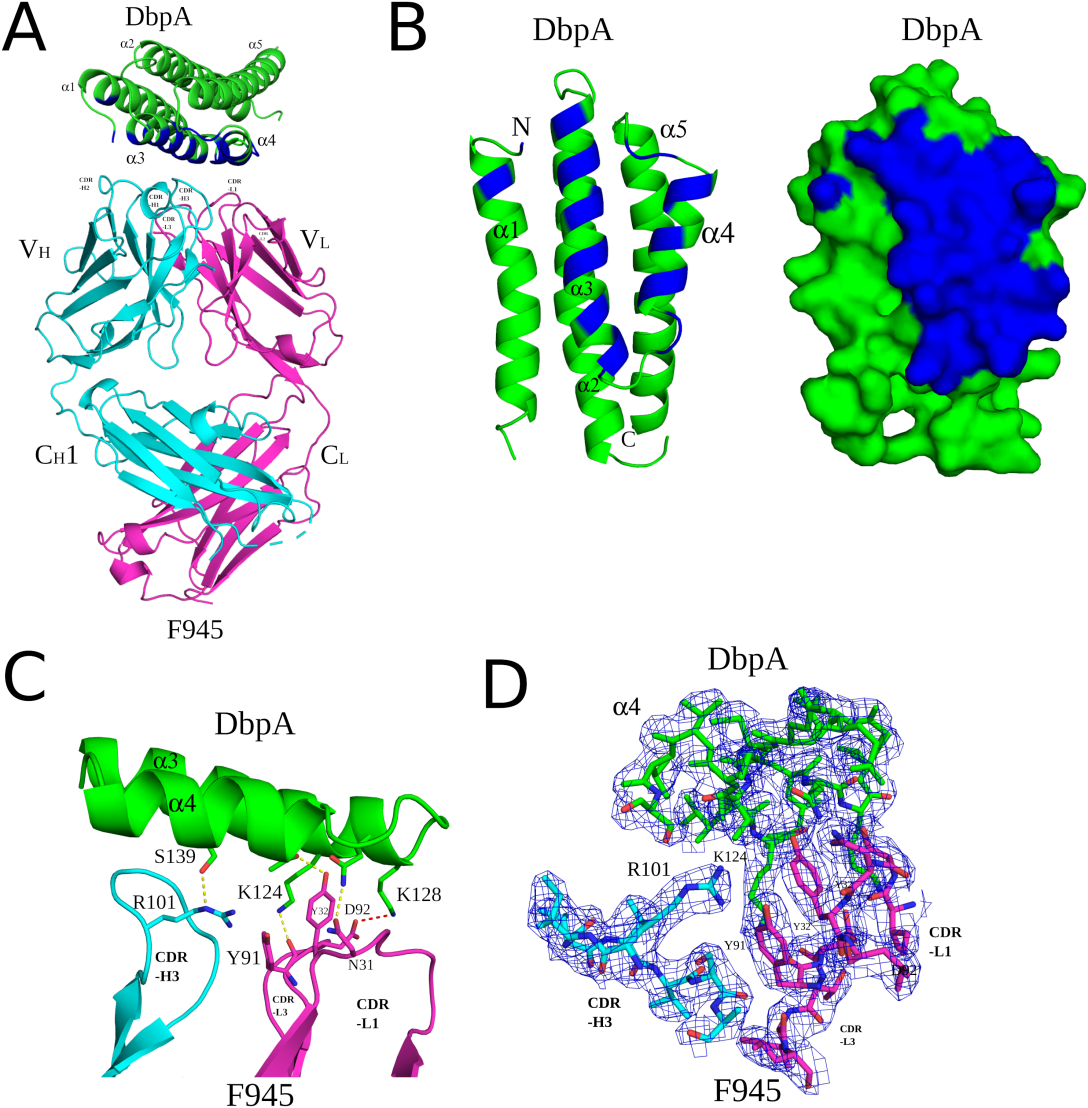
Detailed interactions between F945 and DbpA revealed from the co-crystal structure. **(A)** Ribbon structure of DbpA (green) in complex with a F945 Fab (VH and CH1 elements, cyan; VL and CL, magenta). The DbpA residues that engage with F945 are colored blue. Key secondary structures are labeled (α-helices 1, 2, 3, 4, and 5); **(B)** Ribbon (left) and surface (right) depiction of DbpA (green) with F945-interacting residues shaded in dark blue. DbpA N and C-termini are labeled N and C, respectively. **(C)** Representation of key H-bonds (yellow dashes) and salt bridges (red dashes) between DbpA (green) and F945 VH domain (cyan) and VL domain (magenta). **(D)** The fully refined 2Fo-Fc electron density map at the F945-DbpA interface drawn as sticks with DbpA colored green, the F945 Fab heavy chain colored cyan, and the light chain magenta. The electron density map is presented as a blue mesh at 1.0 σ level. Side chains are drawn as sticks and color coordinated to the main chain color, with nitrogen atoms shaded blue and oxygen atoms shaded red. CDR elements are labeled per convention: CDR-L1, -L3, -L3; CDR-H1, -H2, -H3 **(B)**.

Analyzing the complex, F945 Fab bound DbpA_B31_ in a side-on orientation (**Figures 3**, **4A**). Five of the six F945 CDRs (L1-L3 and H2-H3) made direct contact with DbpA_B31_. The Fab- DbpA_B31_ interface buried a surface area (BSA) of 1,461 Å² and exhibited a high degree of shape complementarity (SC score, 0.63) (**Table 2**). The V_L_ accounted for roughly half (693 Å²) of the total BSA. F945 interacted with DbpA α-helices 3 and 4, as well as the intervening loop (loop 3-4), consistent with the regions of strong protection observed by HDX-MS (**Figure 2**). F945 also engaged DbpA α-helix 1 and its preceding loop (loop 1), as well as the loop between α-helices 4 and 5 (loop 4-5) (**Figure 4B**). Collectively, F945’s epitope forms a distinct patch on the lateral face of DbpA (**Figure 4B**). Moreover, in the context of the bacterial outer membrane, F945 would associate with DbpA in a side-on orientation, assuming DbpA is anchored to the spirochetal outer membrane via its N-terminal lipid moiety and projects perpendicularly away from the cell surface (**Figure 3**). In such a configuration, F945’s V_H_ element would be in close proximity to the outer leaflet of the membrane.

**Table 2:** F945-DbpA interface data.

F945	Total BSA*^a^*	H-bonds^b^	SB^c^	Sc^d^
V_H_, V_L_	1461	9	1	0.63
V_H_	768	1	0	0.70
V_L_	693	8	1	0.61

*^a^* Å^2^; *^b^* hydrogen bonds; *^c^* salt bridges; *^d^* shape complementarity score. Structure submitted to the Protein Data Base PDB ID 9BQW.

The F945-DbpA interface includes 9 hydrogen bonds and 1 salt bridge. The V_L_ domain is responsible for 8 of the 9 hydrogen bonds and the single salt bridge (**Table 2**; **Figure 4C**). The V_H_ domain mediates only a single hydrogen bond between CDR-H3 residue Arg-101 and DbpA Ser-139. For the V_L_ domain, one hydrogen bond is formed between CDR-L1 Tyr-32 and DbpA Thr-135, a second between CDR-L1 Asn-31 and DbpA Gln-127, and a third between CDR-L3 Tyr-91 and DbpA Lys-124. Additionally, a salt bridge occurs between CDR-L3 Asp-92 and DbpA Lys-128 (**Figure 4C**). The failure of F945 to recognize DbpA_297_ is likely due to a Glu at position 128 rather than a Lys as in DbpA_B31_. This residue is not only an important contact point within F945’s epitope but one of the few amino acid polymorphisms between DbpA_297_ and DbpA_B31_ in this region (**Supplementary Figure S1**).

### 3.4 F945 V_L_ germline residues drive extensive interactions with DbpA

**Figure 5:**
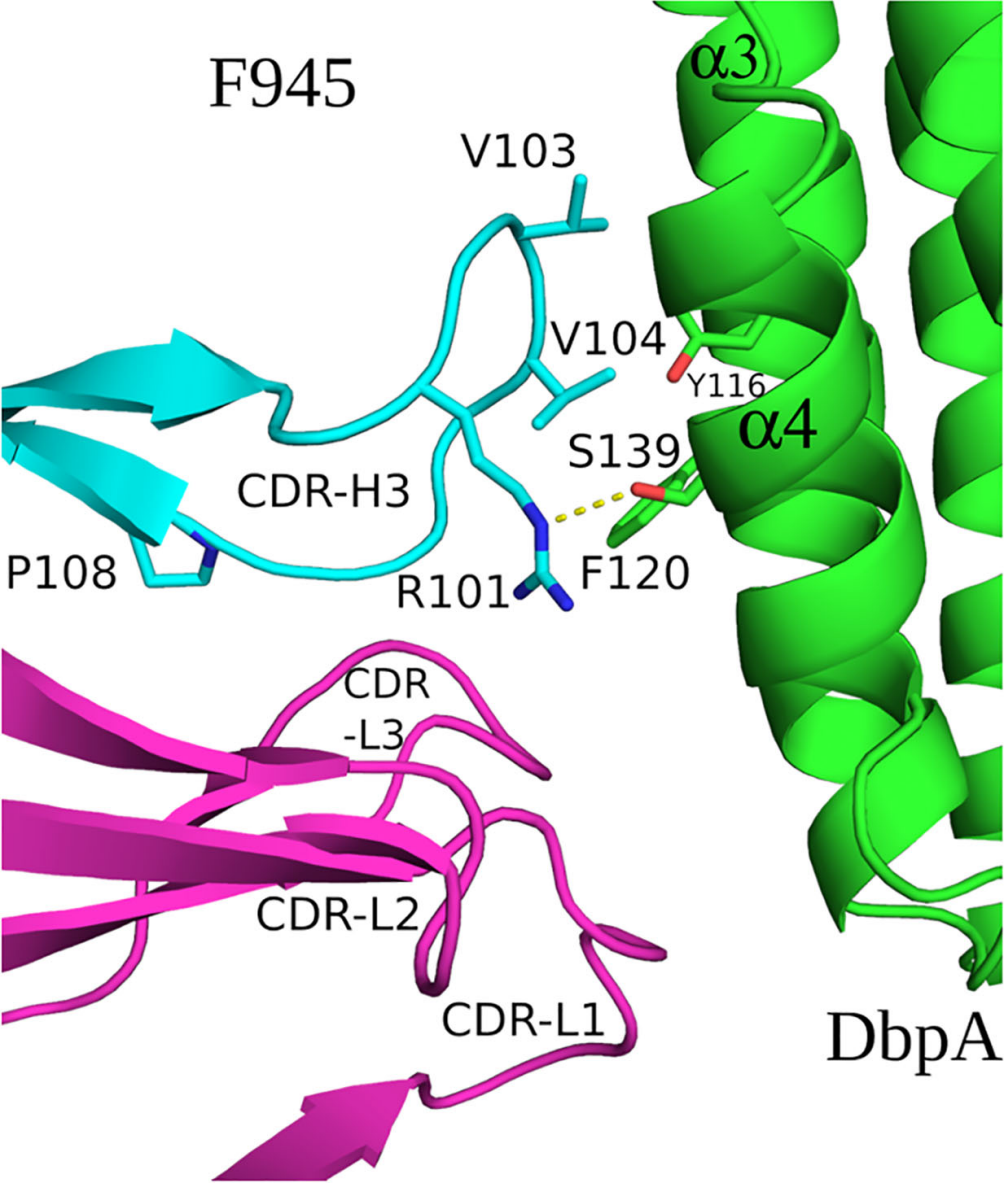
F945 interactions with DbpA. (Top) An alignment between the germline protein sequence of VK1–33 and the F945 light chain. Dots indicate identical residues in F945, while mutated residues are noted. (Bottom) A close-up view of the F945-DbpA interface depicting the configuration of the F945 CDR-H3 that bestows greater connection between the F945 light chain CDRs and DbpA. The F945-DbpA complex is drawn as ribbons with the Fab heavy chain colored cyan, the light chain colored magenta, and DbpA shaded green. CDR-H3 residues Val-103 and Val-104 reveal their hydrophobic interaction with Tyr-116 and Phe-120 from DbpA. The hydrogen bond between Arg-101 and Ser-139 is drawn as yellow dashes. Side chains are drawn as sticks and color coordinated to the main chain color, with nitrogen atoms shaded blue and oxygen atoms shaded red. CDR elements are labeled per convention: CDR-L1, -L3, -L3; CDR-H3.

The extensive interactions between DbpA and the F945 V_L_ CDR elements are unusual and warrant further consideration (57, 58). CDR-L1 plays an oversized role in antigen recognition, forming six of the nine hydrogen bonds between F945 and DbpA and burying 459 Å² of surface area. CDR-L2 and CDR-L3 also contribute to the interaction, forming an additional hydrogen bond and salt bridge, and collectively burying 234 Å² of surface area. The unusually dominant role of F945 V_L_ in antigen recognition is enabled by certain features of the V_H_. Namely, a proline at position 108 causes the H-CDR3 loop to project away from the light chain, thereby creating space for V_L_ to associate with DbpA (**Figure 5**). Second, the conformation of CDR-H3 is stabilized by hydrophobic packing between CDR-H3 (residues Val-103 and Val-104) and DbpA (residues Tyr-116 and Phe-120), along with a hydrogen bond between CDR-H3 Arg-101 and DbpA Ser-139. Collectively, these interactions restrict H-CDR3 movement and reduce conformational entropy, allowing for greater V_L_ engagement with DbpA.

Interestingly, the F945 VL residues responsible for forming hydrogen bonds and the salt bridge with DbpA are all encoded in Vκk1–33 germline (**Figure 5**). Specifically, residues Asp-28, Ser-30, Asn-31, Tyr-32, Ser-67, Tyr-91, and Asp-92 are encoded within the Vκ1–33 germline sequence, per IMGT (51). By contrast, the residues in V_L_ that differ from the Vκ1–33 germline (Q27H, Y49F, N53Y, S76N, P80S, N93T, and P95L) are not within the paratope. Superimposition of the F945 V_L_ from the DbpA-Fab complex with the structure of germline Vκ1–33 [PDB ID 2Q20] reveals a high degree of structural similarity between the two molecules (**Supplementary Figure S5**). This observation has implications in understanding the nature of the humoral immune response to DbpA, because it suggests that the F945 V_L_ may have played a crucial role in initiating recognition of DbpA and in subsequent clonal B cell development, as will be described in the Discussion.

### 3.5 Location of F945’s epitope relative to DbpA’s lysine pocket and flexible linker

**Figure 6:**
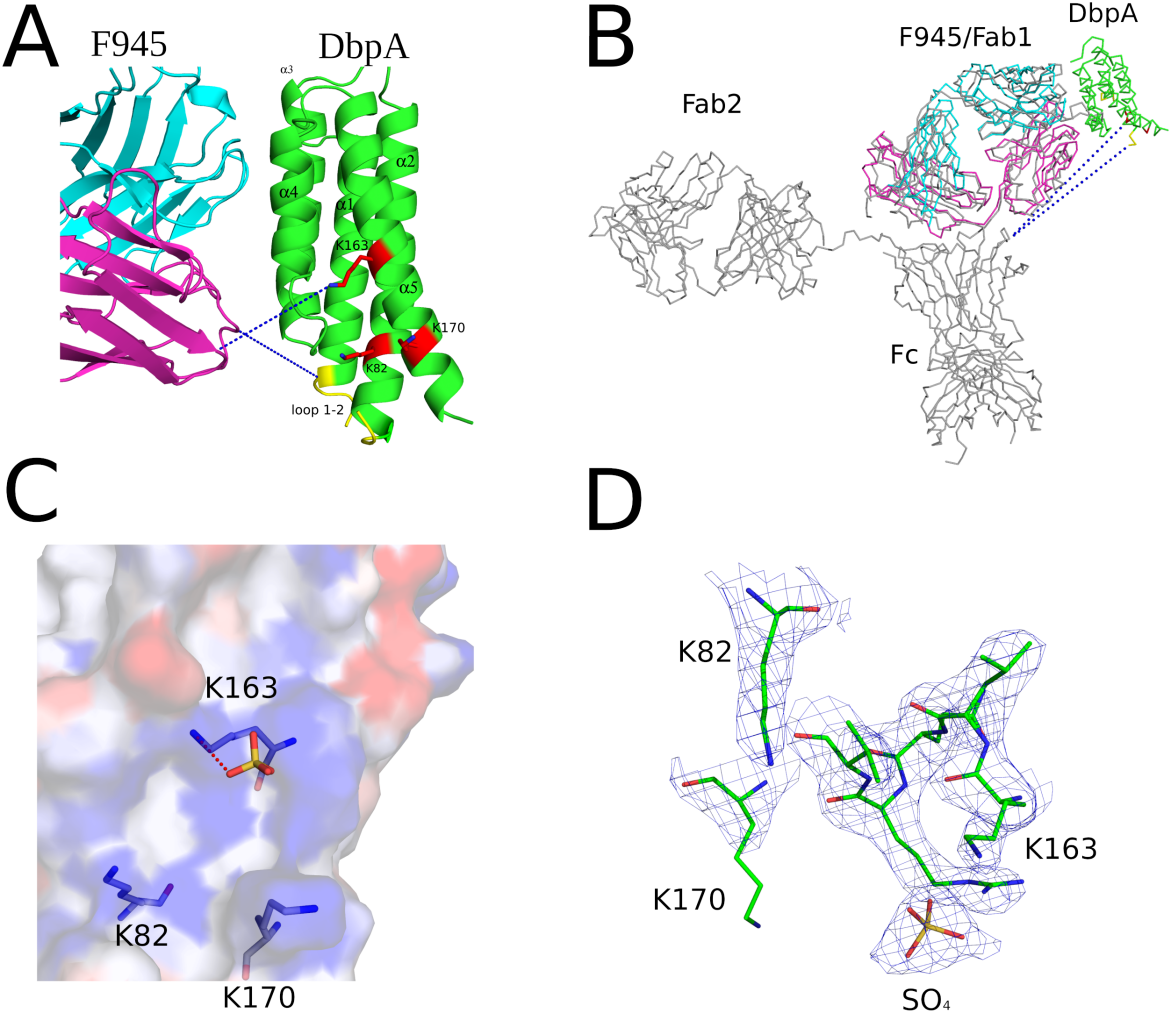
F945 does not directly obstruct known ligand-binding sites on DbpA. **(A)** Close-up view of the F945-DbpA interface (ribbons) showing the considerable distance of ~15Å, depicted as blue dashes, of F945 from known ligand-binding sites on DbpA which included the lysine cluster Lys-82, Lys-163, Lys-170 drawn as red sticks and loop 1–2 colored yellow. This large distance suggests F945 is unable to directly block ligand binding to these DbpA regions. All side chains are drawn as sticks with carbon atoms color coordinated to the main chain color and nitrogen atoms shaded blue. **(B)** The superposed Cα-traces of an IgG1 (PDB ID 1HZH) onto F945-DbpA revealed the Fc region of the superposed IgG1 does not obstruct either of the known ligand-binding sites on DbpA. The closest atoms within the Fc region are over 50Å away from the lysine cluster and loop 1–2 on DbpA. Blue dashes indicate this distance. In panels **(A, B)**, the F945 heavy chain is colored cyan and the light chain magenta with DbpA shaded green. **(C)** The interaction of the known decorin-binding pocket on DbpA lined by Lys-82, Lys-163, and Lys170 drawn as an electrostatic pocket showing the electropositive cavity where the sulfate molecule binds. The electrostatic surface potential map was drawn with the surface color representing electric potential with red color as negatively charged surface, blue color as positively charged surface, and neutral regions colored in white. The salt-bridge between Lys-163 and the bound sulfate molecule is drawn as red dashes. **(D)** The fully refined 2Fo-Fc electron density map at the sulfate-binding site on DbpA with this region of DbpA and the bound sulfate molecule drawn as sticks. The electron density map is presented as a blue mesh at 1.0 σ level. All side chains are drawn as sticks with carbon atoms colored green, nitrogen atoms shaded blue, sulfur colored yellow, and oxygen colored red.

The interaction of DbpA with decorin and other ligands is coordinated by three lysine residues (Lys-82, Lys-163, and Lys-170) that form a pocket associated with substrate recognition (7, 10). F945 Fab does not interact directly with any of these critical lysine residues, nor is it expected to sterically occlude the pocket, as the closest atoms in F945 are located 10-20 Å away (**Figure 6**). F945 is also unlikely to affect the flexible linker between DbpA α-helices 1 and 2 (residues 51-74), a region known to influence heparin sulfate binding (59) (**Figure 6**). Although much of this linker is disordered in the DbpA-F945 Fab structure, the overall position of the loop 1–2 is still ~10-20 Å away from the closest atoms in F945. Moreover, superimposing a full length IgG1 mAb (PDB ID 1HZH) with a mass five times that of DbpA onto the F945 Fab structure reveals that the F945 Fc segment at its closest would be >50 Å from DbpA’s ligand-binding site (**Figure 6B**). That said, we cannot exclude the possibility that the binding a full-length IgG molecule to DbpA on the surface of *B. burgdorferi* affects DbpA’s native orientation and/or membrane display necessary for optimal ligand engagement.

### 3.6 Passive protection studies in a mouse model of *B. burgdorferi* needle infection

**Figure 7:**
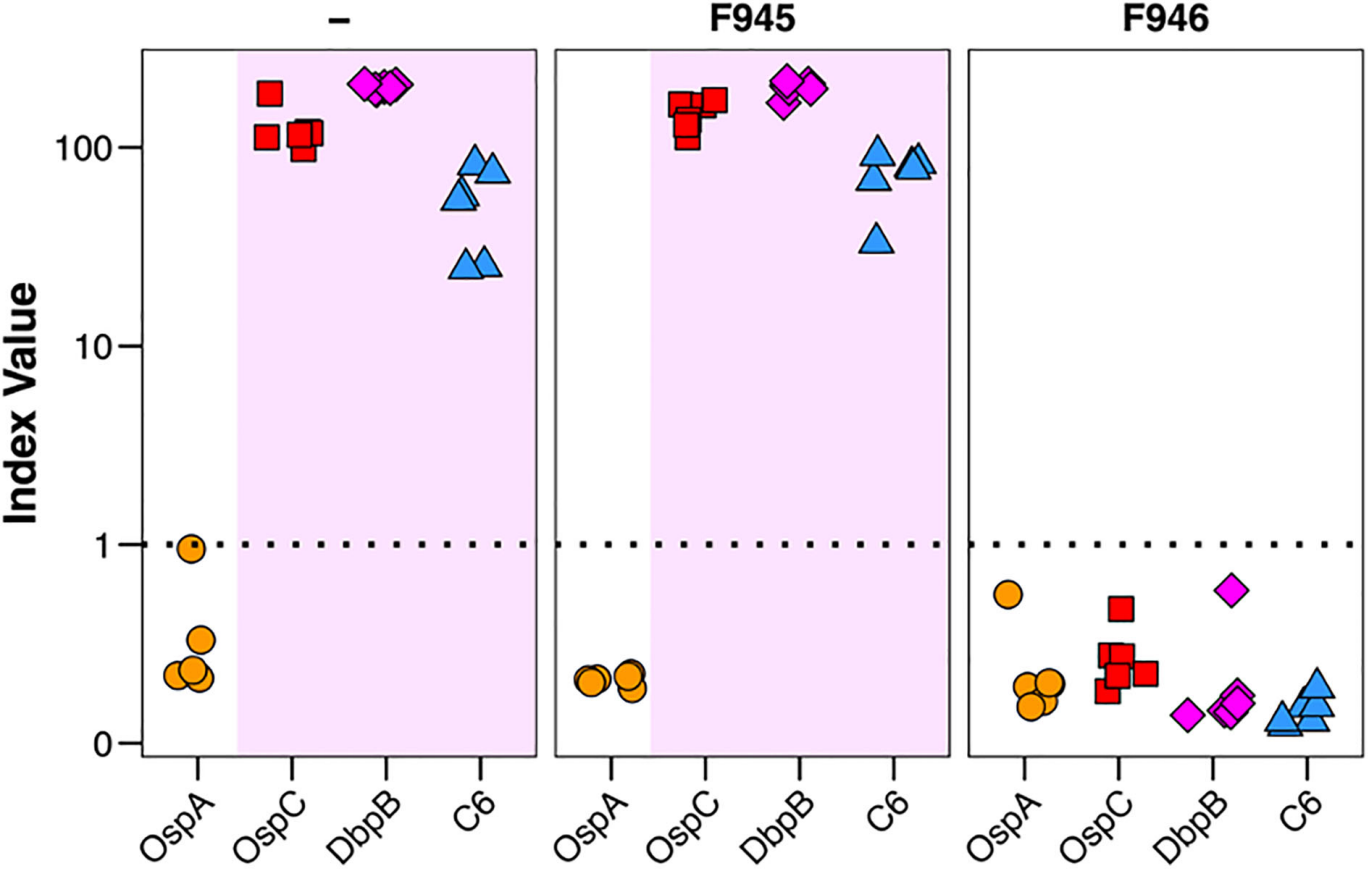
Passive immunity afforded by F945 IgG in a mouse model of needle infection. On study day -1, groups of female BALB/c mice (n=6 per group) were administered F945 IgG1 or F946 IgG1 (anti-OspC mAb) by intraperitoneal injection. A control group that did not receive antibody was also included. On day 0, all three groups of mice were challenged by intradermal injection with 1 x 10^5^ *B*. *burgdorferi* strain B31 A4. Three weeks later, serum samples collected from each mouse and were analyzed by multiplex MIA (Luminex) for the presence of *B*. *burgdorferi* antibodies. The MIA included beads coupled with B31-derived recombinant OspA (circles), OspC (red squares), DbpB (purple diamonds), and VlsE peptide C6 (blue triangles). Seroconversion was assessed as described in the Materials and Methods, with index values >1 shaded in violet and indicate a positive result for the given antigen. Each symbol represents a single mouse.

There are conflicting reports about the role of DbpA antibodies influencing immunity to *B. burgdorferi* infection with some groups observing protection and others not in mouse models (20, 30). To assess F945 in this context, BALB/c mice were passively administered F945 IgG_1_ (120 µg; ~6 mg/kg) via intraperitoneal injection one day prior to being challenged with *B. burgdorferi* strain B31 (1 x 10^5^) by intradermal (needle) injection. As controls, groups of mice received a protective human OspC mAb known as F946 (M. Rudolph, L. Cavacini, D. Vance, and N. Mantis, *manuscript in preparation*) or no antibody at all. A custom multiplex microsphere immunoassay (MIA) that included OspA, OspC type A, DbpB_B31_, and the VlsE C6 peptide was used to assess seroconversion on day 21 post challenge, as described in the Materials and Methods. The six control mice that were challenged with B31 in the absence of mAb pre-treatment treatment all seroconverted as evidenced by antibody titers against OspC, DbpB and C6, but not OspA (**Table 3**; **Figure 7**). The six F945-treated mice also seroconverted with titers virtually identical to the control mice. In contrast, none of the F946-treated mice seroconverted. These results demonstrate that F945 does not protect against *B. burgdorferi* B31 infection in a mouse model but leaves the door open to further investigation into whether F945 protects mice against tick-mediated challenge and/or influences *B. burgdorferi* dissemination kinetics and tissue distributions.

**Table 3:** Passive immunization studies in a mouse model of *B. burgdorferi* needle infection.

mAb*^a^*	Dose (mg/kg)	Seroconversion*^b^*	% Infected
F945	6	6/6	100
F946	6	0/6	0
–	–	6/6	100

^a^mAbs (120 µg; 6 mg/kg) were administered to mice 24 h prior to challenge with *B. burgdorferi* strain B31 A4 by needle injection; *^b^* seroconversion was assessed using a B. burgdorferi multiplex MIA, as described in the Materials and Methods.

## 4 Discussion

Over the past decade, the isolation and functional characterization of human MAbs from convalescent individuals has transformed our ability to decipher the role of specific antigens and epitopes in immunity to complex and antigenically variable pathogens like HIV-1, influenza virus and *Plasmodium falciparum* (60–62). This strategy is only now being applied to *B. burgdorferi* and Lyme disease (4, 37, 53, 63–65). As a case in point, Blum and colleagues cloned V_H_/V_L_ paired sequences from single cell plasmablasts isolated from Lyme disease patients and screened the resulting recombinant MAbs for reactivity with *B. burgdorferi* antigens (4). Not only did that approach result in the identification of a large collection of *B. burgdorferi*-specific MAbs, but it also yielded antibodies with bacteriostatic activities that may contribute to disease resolution (4). In a follow-up study, we characterized the molecular interactions of one such MAb (“B11”) with outer surface protein C (OspC), a *B. burgdorferi* lipoprotein required for early stages of human infection (37).

In this study, we report the first in-depth characterization of a human MAb (“F945”) against DbpA, a highly immunoreactive lipoprotein expressed by *B. burgdorferi* during human infection (3). High-resolution epitope mapping by HDX-MS and X-ray crystallography demonstrated that F945 associates with a lateral face of DbpA in a configuration that is notable for several reasons. First, F945’s primary contacts include DbpA α-helices 1, 3 and 4, but not residues implicated in decorin recognition. Even as a full-length IgG, F945 is not predicted to occlude the three Lys residues (Lys-82, Lys-163, and Lys-170) involved in ligand binding (7, 10). Nor is F945 IgG expected to impact DbpA’s flexible linker between α-helix 1 and 2, which modulates accessibility to the decorin binding pocket (59). Thus, F945 is not expected to impact DbpA-ligand interactions. Efforts to establish quantitative DbpA-ligand interactions are ongoing to enable us to address what impact F945 has on DbpA activity *in vitro*.

Second, F945’s angle of attack is such that it is predicted to associate with DbpA at a right angle relative to the plane of DbpA’s surface. This side-on orientation is striking in its resemblance to how B11 IgG is predicted to associate with OspC (37). However, the DbpA-F945 Fab structure also suggests that the V_H_ element is situated in close proximity to the membrane interface. While the exact membrane topology of lipidated DbpA is unknown (66), it is difficult to envision how F945 IgG accesses its epitope without impinging upon the bacterial lipid bilayer. Perhaps this explains why F945 IgG surface labeling of viable *B. burgdorferi* cells is rather paltry unless the cells are stained immediately upon recovery from frozen glycerol stocks or treated with a mild detergent (e.g., Tween-20) to permeabilize the cell envelope even when DbpA is induced by ectopic expression of RpoS. The issue of surface accessibility was raised years ago by Hagman and colleagues as a possible explanation for why DbpA antisera failed to react with intact *B. burgdorferi* cells *in vivo* (20).

Third, the structure of the DbpA-F945 Fab complex revealed an outsized role for V_L_ germline encoded residues in mediating the DbpA interface. Specifically, the F945 V_L_ accounts for approximately half of the total antigen-antibody buried surface area with DbpA along with eight of the nine H-bonds and the sole salt bridge. CDR-L1 alone contributes ~30% of the DbpA-Fab interface, which is three times higher than the average CDR-L1 contribution (57). Remarkably, all the critical contacts with F945 involve V_k_1–33 germline encoded residues, suggesting that V_k_1–33 containing BCRs are preconfigured to recognize DbpA. While germline recognition of pathogen-associated antigens is not necessarily a new concept (67–69), it is the first example in *B. burgdorferi*. This observation has potentially important implications for understanding the immunodominant and reportedly T-cell independent nature of DbpA (17, 70). Namely, recognition of DbpA by V_k_1–33 BCR elements would potentially lower the threshold for activation of native mature B cells and subsequent clonal B cell development. That said V_k_1–33 recognition of DbpA is likely restricted to certain alleles of *dbpA* (31). For example, F945 binds to DbpA_B31_ but not the closely related DbpA_297_, possibly because of a single Lys/Glu polymorphism at position 128. Thus, the importance of V_k_1–33 BCR recognition in driving the B cell response to *B. burgdorferi* infection remains to be resolved.

While our preliminary studies demonstrated that passively administered F945 was not sufficient to protect mice against *B. burgdorferi* infection, we have yet to investigate a role for F945 in influencing *B. burgdorferi* tissue tropism, retention within specific niches, complement-mediated killing, and spirochete-induced tissue pathology (6, 9, 10, 12–14, 71–73). Nonetheless, it is entirely possible that *B. burgdorferi* is indifferent to F945, due either to the relative inaccessibility of the F945 epitope or the failure of the antibody to occlude the decorin binding pocket. Indeed, the fact that *B. burgdorferi* infection in mice has been shown to proceed unabated in the face of a robust DbpA antibody response would argue for the existence of at least a subset of non-functional antibodies (20, 33). A similar argument could be made in humans, considering that DbpA antibodies are prevalent in Lyme disease patients including those with late disseminated disease (3).

## Data Availability

The datasets presented in this study can be found in online repositories. The names of the repository/repositories and accession number(s) can be found in the article/**Supplementary Material**.
