# Supplementary material for: Germline encoded residues dominate the interaction of a human monoclonal antibody with decorin binding protein A of Borrelia burgdorferi
Source: Front Immunol. 2025 Jul 7;16:1611828. doi: 10.3389/fimmu.2025.1611828 (PMC12277278; doi:10.3389/fimmu.2025.1611828)
Supplement: Supplementary file 1 [file DataSheet1.pdf]

Supplementary Material

1 Supplementary Data

None

2 Supplementary Figures and Tables

2.1 Supplementary Figures

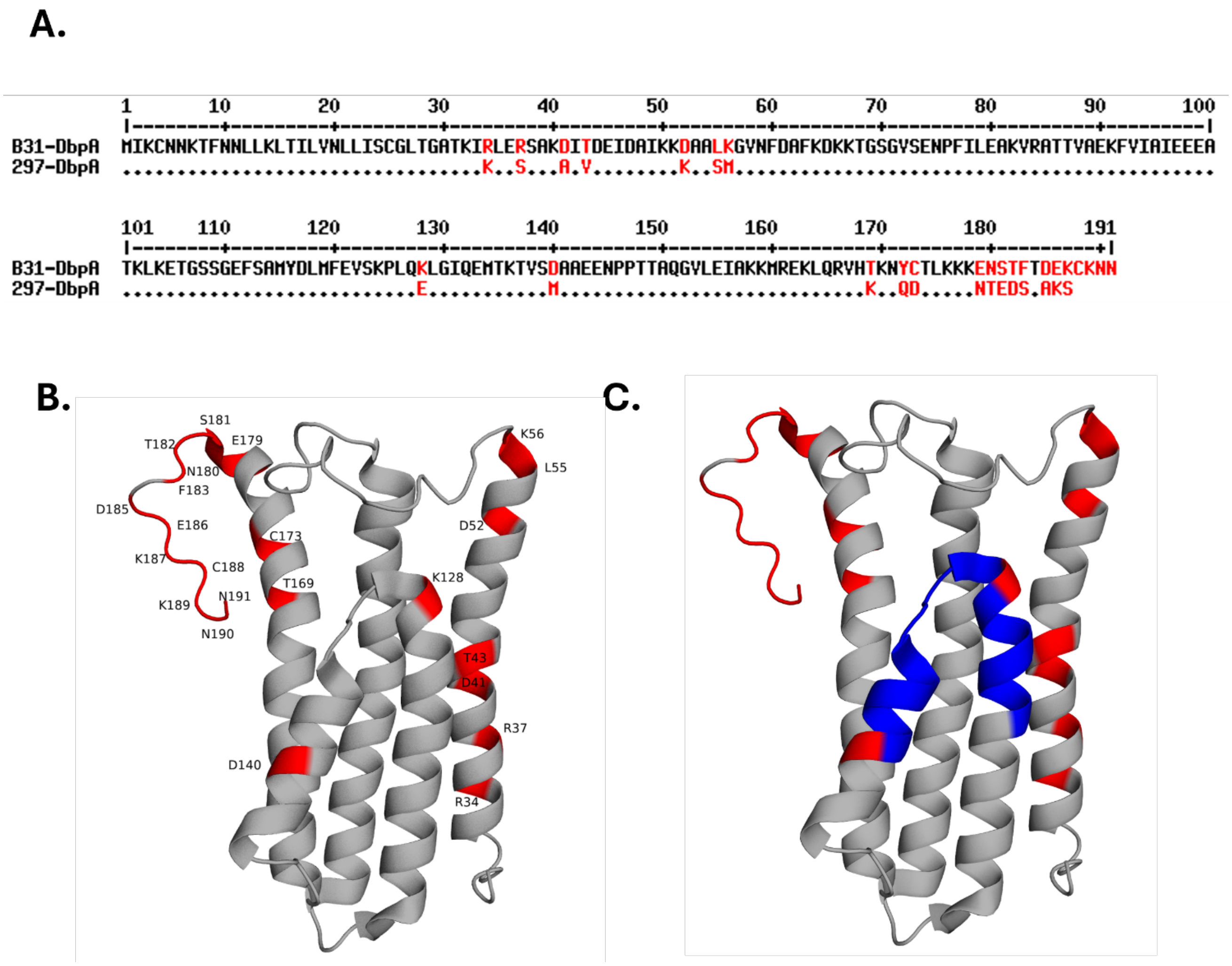

Supplementary Figure 1. Amino acid sequence polymorphisms between DbpA B31 and 297. (A) Amino acid alignment of DbpA from B. burgdorferi B31 (top; UniProt O50917) and 297 (bottom; UniProt A0A9N7AQK9). Polymorphisms are colored red. (B) NMR structure of DbpA B31(PDB ID

2LQU) with polymorphisms as shown in panel A highlighted in red and labeled. (C) The PyMol image from panel B with the addition of F945's epitope colored blue.

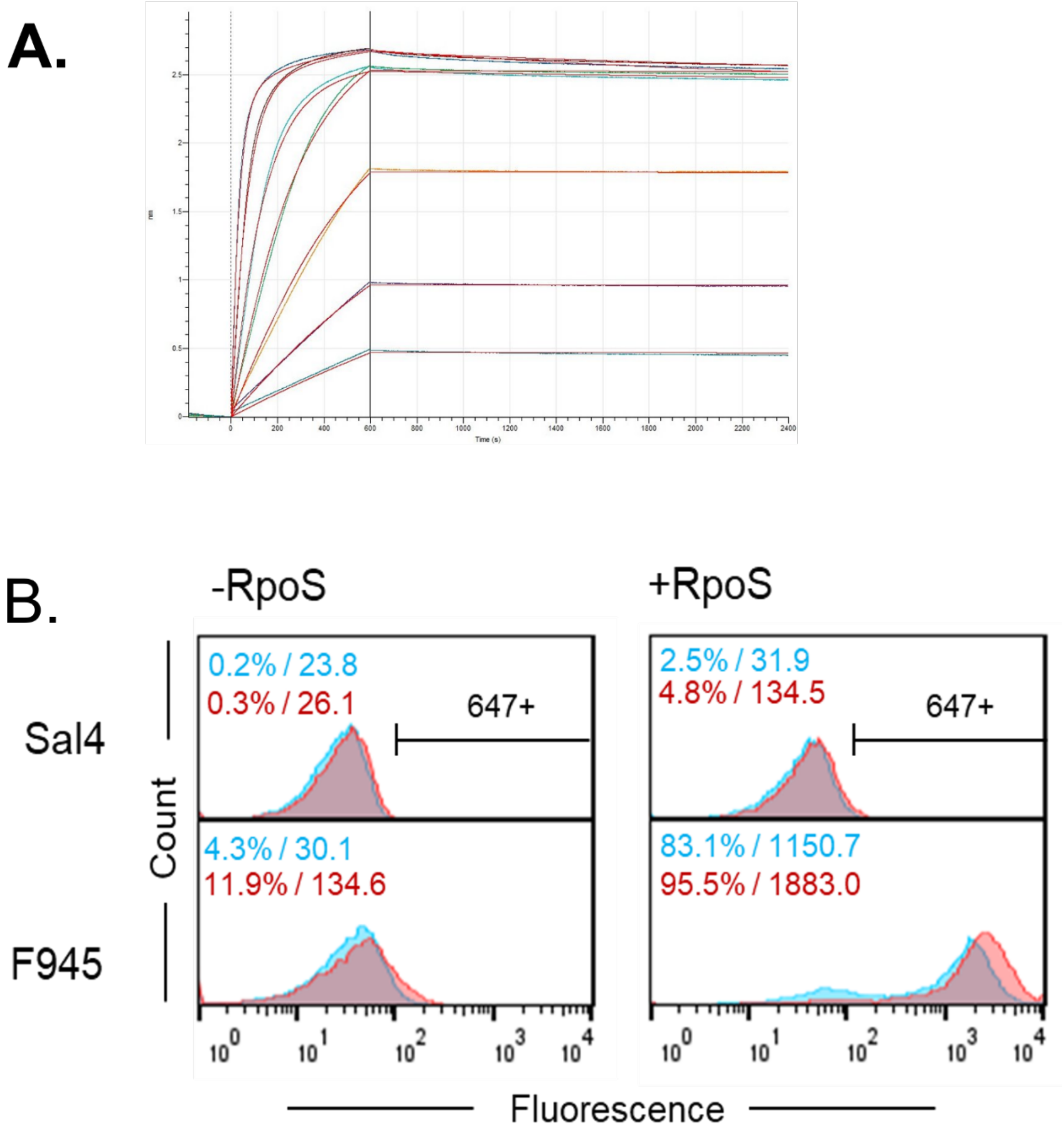

Supplementary Figure 2. F945 reactivity with DbpA. (A) Biolayer interferometry sensorgrams of F945 binding to Avi-Tagged (and biotinylated) DbpA captured on streptavidin sensors. Concentrations range from 100 nM F945 to 1.56 nM and are fit to a 1:2 bivalent analyte binding model. (B) Flow cytometric analysis of F945 IgG reactivity with the surface of B. burgdorferi strain GGW941 with RpoS uninduced (-RpoS; left panels) or induced (+RpoS; right panels) and not treated (blue) or treated (red) with 0.1% Tween-20. The Salmonella-specific mAb, Sal4 IgG, was used an isotype control (top panels). For the histograms, the Y-axis refers to number of events ("count") and

the X-axis is fluorescence intensity ("fluorescence"). The numbers in the top left corner of each box represent positive events (% of total) and median fluorescence intensity (MFI). The horizontal bar to the left of the histograms in the top panels indicate threshold for positivity. Each panel depicts a single experiment representative of 2-3 biological replicates.

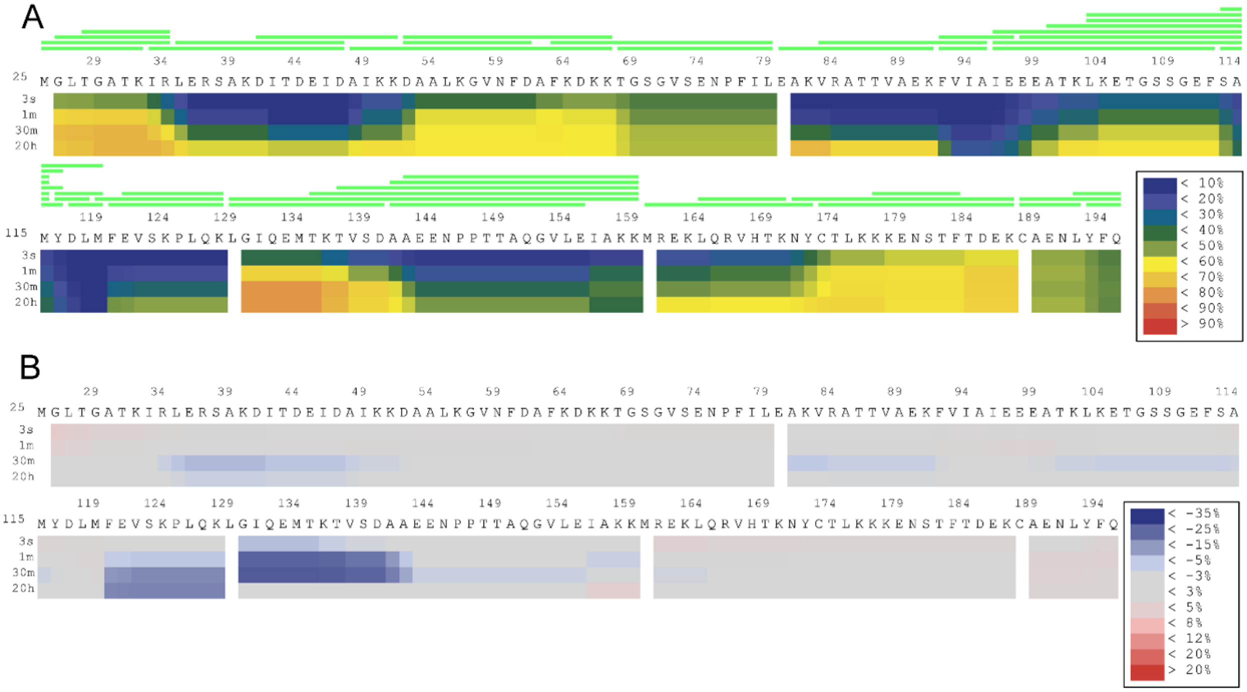

Supplementary Figure 3. DbpA-F945 HDX-MS analysis. (A) Coverage map and exchange profile of unliganded DbpA B31. Green bars above the primary sequence indicate observable peptides used for extracting HDX-MS data. Colors from blue-green-yellow-red indicate extents of exchange across the sequence measured at the 4 time points. (B) Difference in exchange between unliganded and F945-bound DbpA B31 across the full sequence. Regions in blue and red indicate decreased or increased exchange in the F945 complex, respectively.

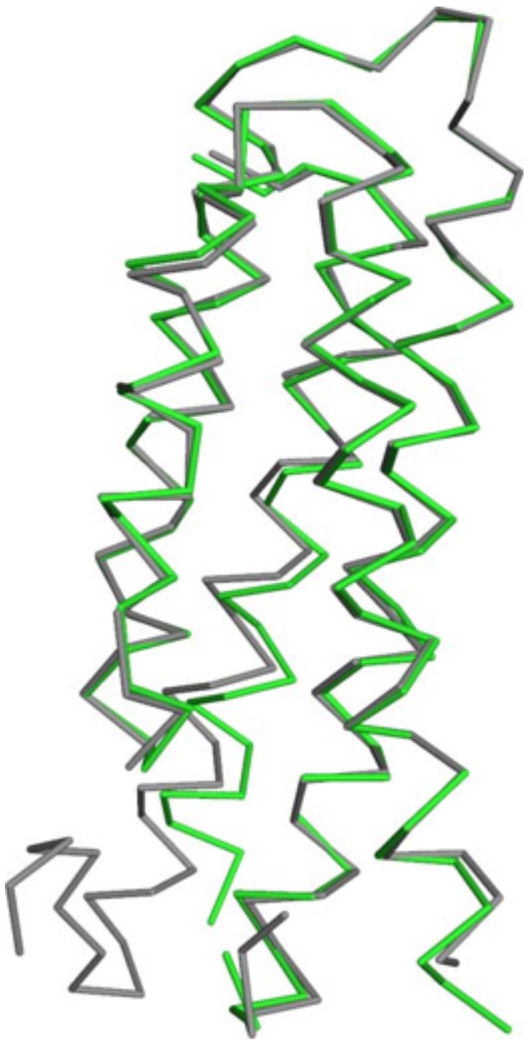

Supplementary Figure 4. DbpA tertiary structure is unchanged following F945 binding. Drawn are the super positioned Cα-traces of DbpA (green) from the F945-DbpA B31 structure and unliganded DbpA 297 (PDB ID:40NR) (dark grey) depicting their structural similarity.

A

```
           1        10        20        30        40        50        60        70        80        90   95
           |--------+---------+---------+---------+---------+---------+---------+---------+---------+----|
  VK1-33   DIQMTQSPSSLSASVGDRVTITCQASQDISNYLNWYQQKPGKAPKLLIYDASNLETGVPSRFSGSGSGTDFTFTISSLQPEDIATYYCQQYDNLP
 F945_VL   ..........................H......................F..Y.......................N..S............T.L
```

B

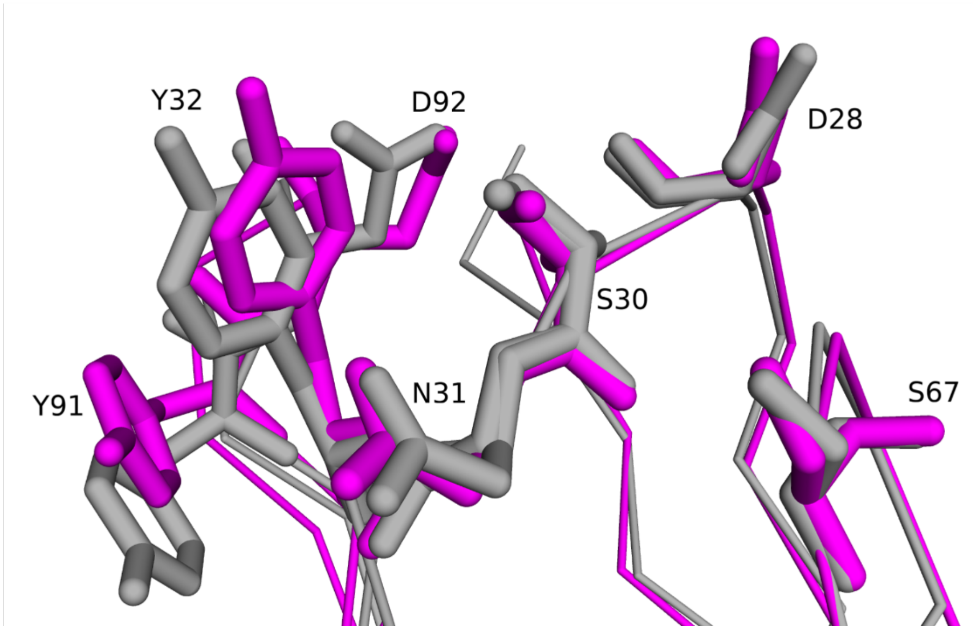

Supplementary Figure 5. Similarity of F945 VL with germline. (A) Amino acid alignment of the human germline light chain kappa gene VK1-33 with F945's light chain V region. Polymorphisms are colored in red. (B) F945's light chain (magenta) and the VK1-33 germline (gray, PDB ID 2Q20) are shown in ribbons with F945's seven paratope residues that form the H-bonds and salt bridge with DbpA depicted as sticks and labeled with single amino acid abbreviation and residue number. The seven paratope residues align with an RMSD of 0.353 A, while the entire light chains align with an RMSD of 0.448. None of these seven residues are mutated from the germline gene (see panel A). Taken together, this suggests that the residues most important for binding to DbpA are not only present in the germline precursor but spatially preconfigured to recognize DbpA.

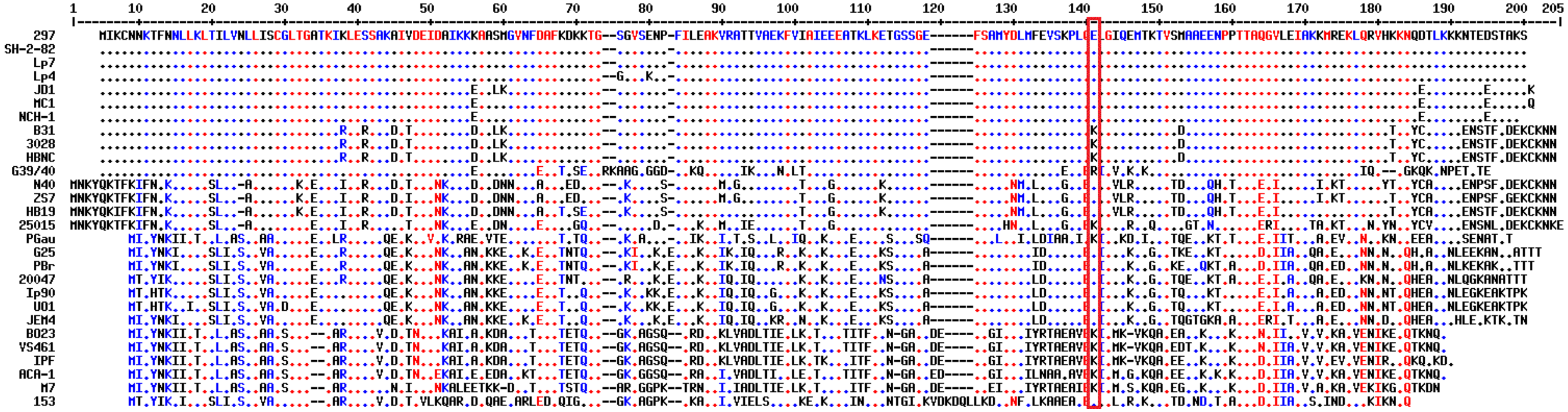

Supplementary Figure 6. Multiple amino acid sequence alignments of DbpA. Amino acid multiple sequence alignment of DbpA from several different species and strains of Borrelia. The residue at position 141 is boxed in red and represents the mutation in the F945 epitope region between DbpA B31 (lysine) and 297 (glutamic acid) likely responsible for F945's differential binding. Note that due to gaps in the alignment, this residue is not residue 141 in DbpA B31, but residue 128 (see Figure S1A).

2.2 Supplementary Tables

Table S1. **F945 VH and VL sequences**

VH	QVQLVESGGGVVQPGRSLRLSCAASGFSFNTYAFHWVRQGP GKGLEWVAGISFDGSKRYYADSVKGRFTVSRDNSKNTLYLQ MNGLIPEDTAVYYCARDRRIVVVSAPGYWGQGTLVTVSS
VL	DIQMTQSPSSLSASVGDRVTITCQASHDISNYLNWYQQKPGK APKLLIFDASYLETGVPSRFSGSGSGTDFTFTINSLQSEDIATY YCQQYDTLLSFGGGTRVEIK
